# Bisphenol A—What Do We Know? A Global or Local Approach at the Public Health Risk Level

**DOI:** 10.3390/ijms25116229

**Published:** 2024-06-05

**Authors:** Angelika Edyta Charkiewicz, Wioleta Justyna Omeljaniuk, Jacek Nikliński

**Affiliations:** 1 Department of Clinical Molecular Biology, Medical University of Bialystok, 15-269 Bialystok, Poland; 2 Department of Analysis and Bioanalysis of Medicines, Medical University of Bialystok, 15-222 Bialystok, Poland

**Keywords:** bisphenol A, toxicity, sources, biomonitoring, risk assessments

## Abstract

BPA has demonstrated enormous multisystem and multi-organ toxicity shown mainly in animal models. Meanwhile, the effects of its exposure in humans still require years of observation, research, and answers to many questions. Even minimal and short-term exposure contributes to disorders or various types of dysfunction. It is released directly or indirectly into the environment at every stage of the product life cycle, demonstrating its ease of penetration into the body. The ubiquity and general prevalence of BPA influenced the main objective of the study, which was to assess the toxicity and health effects of BPA and its derivatives based on the available literature. In addition, the guidelines of various international institutions or regions of the world in terms of its reduction in individual products were checked. Bisphenol A is the most widely known chemical and perhaps even the most studied by virtually all international or national organizations, but nonetheless, it is still controversial. In general, the level of BPA biomonitoring is still too high and poses a potential threat to public health. It is beginning to be widely argued that future toxicity studies should focus on molecular biology and the assessment of human exposure to BPA, as well as its substitutes. The effects of its exposure still require years of observation, extensive research, and answers to many questions. It is necessary to continue to deepen the knowledge and interest of many organizations, companies, and consumers around the world in order to make rational purchases as well as future choices, not only consumer ones.

## 1. Introduction

In recent decades, the xenoestrogen bisphenol A (BPA; 4,40-isopropylidenediphenol) and selected parabens have gained scientific attention due to their highly adverse health effects [1,2]. Bisphenol A is an organic synthetic compound (chemical formula—(CH_3_)_2_C(C_6_H_4_OH)_2_) with a molecular weight of 228.29 g/mol. In 1891, Alexander P. Dianin first combined phenol with acetone in the presence of an acid catalyst. In contrast, it was not synthesized until 1905 by Theodor Zincke of the University of Marburg in Germany. The reaction of BPA with phosgene (carbonyl chloride) in the 1950s produced a transparent hard resin known as polycarbonate, which has found wide and widespread use [3,4,5]. This organic compound is white in color, is mostly solid in the environment, and has a mild phenolic odor [6].

There are six best-known bisphenol derivatives (BPA, BPF, BPS, BPAF, bisphenol E (BPE), and bisphenol B (BPB)), with each appearing to be equally toxic and not very biodegradable at the present time [4]. However, Nowak and Jakopin have reviewed the following list of bisphenols: BPA, BPAF, BPAP, BPB, BPC, BPC2, BPE, BPF, BPFL, BPG, BPM, BPP, BPP, BPP, BPP, BPS, BPTMC, BPZ, dinitrobisphenol A (DinitroBPA), and bisphenol A diglycidyl diglycidylase, and converted fluorinated products, concluding in their study that quick insights into their respective toxicological profiles should be made [7].

It is worth noting that the compound’s half-life in both soil and water is up to 5 days, while in air it is only 1 day [8,9,10].

Estimates in 2015 showed annual production of up to 4.85 million tons [4]. Its global market in 2022 was about 5600 tons, while forecasts to 2032 predict an increase of 3.51% (to 8000 tons). The current global leader in terms of consumption is the Asia Pacific region (55% of the market), followed by Europe and North America. Meanwhile, in production, Asia Pacific accounts for 35% [5,11,12].

As BPA can be released directly or indirectly into the environment at any stage of a product’s life cycle (e.g., during production, consumption, or disposal), it is worth noting its toxicity and ease of penetration into the body [13,14]. The fastest and easiest route for its release into beverages or food products is heating under alkaline or acidic conditions, which effectively accelerates its absorption into the human body [3,15,16]. BPA is more rapidly released from various types of polycarbonates due to UV, heating, and aging of the material [9]. It is very soluble in fats, but is less so in water [13,17].

They have been widely used in medicine for more than 80 years [1,18]. Its uncanny ability to interact with many systems can occur many years after exposure, due to its accumulation. Its content in selected products, sometimes intended for children, ranges from 9.2 to 62.7 µg/mg, while globally it is supplied up to 30.76 ng/kg/day [1,4,19,20,21,22,23,24]. The most common sources of exposure are through direct skin contact, inhalation, oral ingestion, and sometimes from the air. As its adverse health effects have been repeatedly confirmed, it is necessary to identify the most important sources of exposure [10,14,24,25,26,27,28]. Many times, the magnitude of exposure is related to the use of hygiene measures, which may result from individual patterns, physiological characteristics of the body, and socioeconomic and demographic characteristics, such as the amount and frequency applied to selected body sites, season of use, frequency of inhalation [20,27,29].

The ubiquity and general prevalence of BPA influenced the main objective of the study, which was to assess the toxicity and health effects of BPA and its derivatives based on the available literature. In addition, the guidelines of various international institutions or regions of the world in terms of its reduction in individual products were checked.

The following manuscript offers a unique aspect, presenting the multidirectional impact of bisphenol in terms of public health; general health, or lack thereof; environmental impact; and more importantly, institutional as well as individual actions, or lack thereof, in terms of daily choices.

## 2. Health Effects

**Figure 1:**
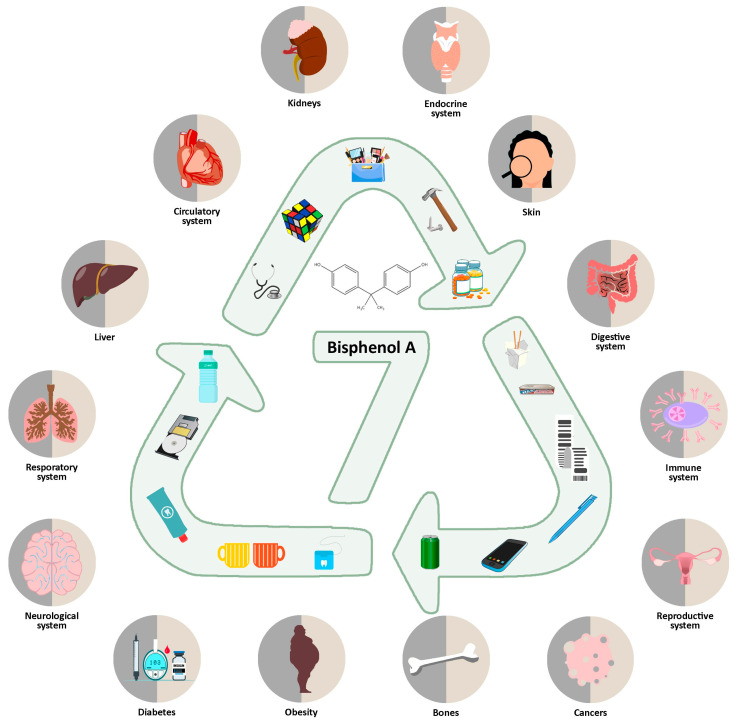
Sources and impact of bisphenol A on the human body.

The first risk assessments were started in 1936 by Dodds and Lawson [18], while a full, complete study containing the health effects of BPA was not really analyzed in depth until 2006, at which time EFSA and its assessment panel returned a full report [30]. Evidently, a number of studies suggest that BPA has toxic effects and its half-life in the body is less than 6 h. Meanwhile, its variant (isotopic BPA-d16) derived from diet is eliminated in the urine in up to 24 h, is absorbed through the skin, and persists for up to a week [10,13,31,32]. Typically, BPA concentrations in the blood range from 0.2 to 20.0 ng/mL [9,33]. The main route of exposure is through diet. Bisphenol A (BPA) has been shown to be associated with estrogenicity and endocrine-disrupting properties, has adverse neuropsychiatric effects and may be involved in the formation of NETs; it may be indirectly involved in miscarriage, cardiovascular disease, especially hypertension, reduced heart rate variability, diabetes, obesity, eye disease, allergic skin reactions, respiratory irritation, acute/chronic kidney disease, and liver disease (Figure 1). However, the effects of BPA depend on the dose, the sex of the patient, the tissue and developmental stage of the exposed tissue/organ, and the time of exposure [14,18,19,21,24,26,28,29,31,33,34,35,36,37,38,39].

Its exposure also contributes to disorders or various dysfunctions in the tissues associated with these diseases and various cell signaling pathways [28,37,40]. Because of its properties, BPA also has the potential to affect cancer formation and impair reproductive function, including in men, through binding to androgen receptors [3,13,14,24,41]. 

According to the World Health Organization, there is no conclusive evidence that BPA affects immune function [13,42]; meanwhile, EFSA has confirmed its negative effects [7,14]. BPA is most commonly detected in blood (including umbilical cord, menstrual), urine, breast milk, hair, adipose tissue, amniotic fluid, or semen [12,13,28,39]. BPA concentrations in urine can be determined using a variety of methods, such as mass spectrometry (MS), HPLC, radioimmunoassay (RIA), immunoenzymatic immunosorbent assay (ELISA). It can be determined with the highest sensitivity with the following methods: tandem HPLC mass spectrometry (LC-MS/MS) and gas chromatography–mass spectrometry (GC-MS) [43].

### 2.1. The Digestive System

It is worth noting that the gastrointestinal tract is unquestionably its largest source of absorption [13,24,44]. BPA is fairly rapidly absorbed in the gastrointestinal (up to a few hours) tract and then metabolized to BPA-glucuronide (BPA-gluc) via coupling with UDP-glucuronic acid in the intestines and liver and excreted in the urine or feces. Both water and food are major sources of BPA exposure [10,13,16,27]. It is noteworthy that in this case the intestines are particularly susceptible to the adverse effects of this xenoestrogen. The pathomechanism of its action is still unknown, if only by positively affecting serotonin (5-hydroxytryptamine, 5-HT) cells in the intestinal donut layer and their 5-HT function. Meanwhile, its very correlation with 5-HT levels is wrongly associated with the central nervous system, where almost 95% of the body’s serotonin is found in the gastrointestinal tract [45,46]. A 2017 study by Górecki et al. found that average dietary exposure to BPA was 0.048–0.050 μg/kg bw/day for children and adolescents aged 3–17 years, 0.034–0.035 μg/kg bw/day for adults, and 0.047–0.049 μg /kg bw/day for pregnant women [47]. It should be noted that infant exposure to BPA is related to the concentration of BPA in breast milk, mainly deposited in the fat of the mammary glands. Thus, the average amount of BPA is 0.3 μg/kg bw/day in exclusively breastfed infants (0–6 months) and decreases after the introduction of solid foods [13,48]. Unfortunately, BPA may contribute to altering the composition of the intestinal microbiota, the complex polymicrobial ecosystem colonizing the gastrointestinal tract, thus disrupting the host’s metabolic processes [49].

Although, there are still many aspects related to the negative effects of BPA on the gut that remain unclear and require further observations.

### 2.2. Respiratory System

The second source of BPA absorption in the general population is the respiratory tract. Indoors, its concentration can be as high as 9.62 × 10^−4^ µg/kg bw/day (78% of non-dietary exposure). It can migrate into the air during the manufacture of products containing synthetic BPA polymers as well as during use. Household items made with it (e.g., epoxy-based flooring, adhesives, electronic equipment, and printed circuit boards) can release and volatilize toxic BPA during long-term use. As it can unfortunately accumulate in dust, it can be inhaled, ingested, and consequently absorbed by the human body [7,13]. Unfortunately, air and dust are also major routes of exposure for some worker populations, especially in the processing sector where BPA is regularly used [13].

It is still unclear how it can affect the cells of the respiratory system, particularly the trachea and lungs. As of now, there are limited studies involving the effects of this xenoestrogen on lung fibroblasts. Frequent exposure induces bronchial eosinophilic inflammation and frequent allergic sensitization, sometimes associated with chronic inflammation, DNA damage, oxidative stress, cell apoptosis and necrosis, multiple organ incarceration, etc. All symptoms are dependent, as usual, on the time and amount of exposure [16].

A study on a Chinese population (615 NSCLC patients and healthy controls each) examined between 2016 and 2018 at Tongji Hospital of Science and Technology (HUST) in Wuhan showed that its levels were significantly higher in patients with non-small cell lung cancer [50]. The alveolar cells of the lungs are definitely altered with prolonged exposure to it, and worse, it can harm the respiratory system in children. BPA also leads to morphological changes and additionally induces metastasis of lung cancer [16,51]. Unfortunately, BPA can frequently alter the function of lung cells leading to changes in their appearance and movement [16,31,39].

There are many studies confirming endocrine disruption, although more reliable and long-term studies confirming the effects on the respiratory system are still largely lacking.

### 2.3. Reproductive System

BPA may have a significant association with the reproductive system, both in men and women [10,13,24,31]. BPA is distributed throughout the body, crossing the blood–brain barrier and the placenta [32].

In their studies, Liu et al. have repeatedly suggested a link between this xenoestrogen and sexual dysfunction in men, reduced semen quality, impaired male genital development, elevated prolactin (PRL) levels or even fertility regulation in men [12,24,52]. Meanwhile, studies of men from 2014 to 2015 reporting on fertility treatment yielded BPA concentrations in the range of 0.38–21.93 ng/mL in blood, showing negative correlations between its levels and sexual desire, erectile capacity and ejaculatory intensity, or overall sexual function [53]. Other studies also suggest negative effects on testicular function, which can occur even in the fetal or early postnatal period [12,24,31,54]. It is also hypothesized that BPA may have deleterious effects on spermatogenesis and steroidogenesis, sperm concentration, as well as total sperm count [55,56]. By interfering with sex hormones, it affects more pronounced development during the fetal period of the male reproductive system [24,56]. It can also increase the levels of etheradiol (E2), progesterone, serum luteinizing hormone (LH) and testosterone (T) in men, decrease serum cortisol, and more importantly, damage sperm [57,58].

Meanwhile, Omeljaniuk et al. showed that in women with spontaneous miscarriage, BPA concentrations (27.3–39.72 ng/mL) were significantly elevated in the serum of women who had a miscarriage compared to the control group (3.5 ng/mL). Thus, it may play an important role in miscarriage through the formation of neutrophil extracellular traps (NETs). The authors also suggest that BPA exposure should be strongly limited in women undergoing treatment and those suffering from recurrent miscarriage [19]. Similar conclusions about the high risk of recurrent miscarriage (RM) and its impact are supported by other researchers [59]. Interestingly, Özel et al. showed increased BPA levels (8.45 ± 4.2 ng/mL) in diagnosed women with primary ovarian insufficiency (POI) [60]. In a study of women with polycystic ovary syndrome (PCOS) working in conditions of high BPA exposure, BPA levels were higher than in healthy women, confirming its potential role in PCOS pathophysiology [61]. A 7-year (1997–2004) cohort study of pregnant women in Mexico and their children [62], as part of the Early Life Exposure in Mexico to Environmental Toxicants (ELEMENT) project, strongly suggest that aspects of reproductive development may be affected by exposure to this estrogen. The authors also emphasize that samples should be analyzed at each trimester, as well as throughout pregnancy, with the goal of maintaining health in populations ripe for reproduction [62].

Results from human placenta, peripheral blood, and urine collected from women with pregnancies diagnosed with FGR (fetal growth restriction) (n = 12) and healthy control pregnancies (n = 12) at Tongji Medical College, Huazhong University of Science and Technology (Wuhan, China) from June 2022 to July 2023 provided new information on the developmental toxicity of BPA in placental trophozoa, which may provide a basis for the prevention and treatment of BPA-related diseases [31].

A study evaluating 700 Chinese couples between 2013 and 2015 found that any increase in urinary BPA was associated with a 13% reduction in fertility and a 23% increase in infertility risk [63]. Another study also confirms the risk of its concentration with premature birth, this time among Mexican women [64]. Information including its effect on premature rupture of membranes (pPROM) is also of concern [13]. Meanwhile, serum levels of BPA in pregnant women may be positively related to the risk of preeclampsia [13,18,65].

There are still insufficient studies and evidence evaluating the effects of BPA on fertility in healthy women as well as men.

### 2.4. Development and Its Potential Disorders 

There are now studies confirming the potential impact of BPA on offspring development as well as its prenatal impact [13,49,66]. Along with its increased exposure, shorter anogenital distances (AGD) in the first trimester of pregnancy are also observed [67]. As some researchers suggest, its exposure already begins in utero and can unfortunately continue even into the post-breastfeeding period [68]. Additionally, toxic effects for BPA have been documented in neonatal intensive care unit exposures, through various types of contact with medical equipment containing it [18].

Additionally, in school-aged girls, there is a strong association with idiopathic central precocious puberty (ICPP) [69]. The CHAMACOS cohort study showed that BPA can also delay sexual maturity in girls, while accelerating it in boys [70]. In contrast, general trends tend to report accelerated development and maturation in girls, mainly through accelerated uterine development [62]. Meanwhile, a study of urinary BPA concentrations in 300 children (INMA “Environment and Childhood” ages 9–11, Spain) showed that the maximum exposure to this xenoestrogen can affect the behavior of the youngest, especially boys [66].

These bisphenols, by mimicking some of our hormones, can affect sex-specific development. In women, by inducing changes in estradiol E2, it disrupts early puberty, affects abnormal menstrual cycles, and worse, can interfere with gonadotropin fertility treatment and increase the likelihood of endometriosis [18,24].

BPA exposure in pregnant women can also be followed by low birth weight, problematic prosocial behavior, anxious and depressive behavior, numerous sleep-related problems, the very development of language in offspring, emotional control and inhibition, or aggressive behavior syndromes, among others [66,71,72]. BPA, by interfering with oxidative stress and affecting mitochondrial dysfunction, is likely a risk factor for autism [13], impaired memory function or short-term memory difficulties [33], and attention problems [66]. It can interfere with normal brain development and later behavioral patterns related to social and non-social behavior, persisting into adulthood [66,73]. A study of 346 couples (mother–child, 2003–2006) from Cincinnati, OH from pregnancy to age 8 found that BPA was associated with more externalizing behavior, persisting for up to 2–8 years [73].

Unfortunately, there is still a lack of extensive and conclusive studies in humans showing the effects of this xenoestrogen on brain health and neurological development [74]. As mental and behavioral problems in children are now a very serious public health problem, it is imperative to pay attention not only to their increasing frequency but also to their exposure to all environmental factors, including bisphenol A.

### 2.5. The Immune System

Undeniably, BPA is correlated with immune function, inflammation, and oxidative stress. Laboratory conditions confirm that it can induce mitochondrial damage or cell apoptosis. By disrupting the balance of the antioxidant system, it can affect, among other things, the down-regulation of antioxidant gene expression and the formation of lipid peroxidation (LPO) [13,24,31]. BPA, by affecting a change in gene transcription, disrupts the immune system through, among other things, the expression of sex genes. Thus, it induces an autoinflammatory response and deregulation of immunoglobulin T-reg [24,74]. It may also contribute to other mechanisms that promote autoimmune expression and progression [74].

BPA is considered to be potentially implicated in DNA damage caused mainly by its single strand breaks, causing impairment of genomic integrity during meiosis (severely affecting the development of the organism and offspring), inhibition of DNA-dependent protein kinase (DNA-PK) activity and telomerase activity, modification of purines [13,31,75,76]. To date, the following main three types of epigenetic modifications have been discovered: DNA methylation, histone modification, and non-coding RNA regulation [12,13,24,74].

BPA toxicity may be relevant to molecular changes at the molecular level (exosomes and ncRNAs such as circRNAs and long non-coding RNAs). Currently, high-throughput sequencing and gene chips are effective tools to study the molecular mechanism of its toxicity and other xenobiotic compounds. As there are nevertheless few studies on the mechanism of BPA with the above-mentioned methods, there is a strong need to find a golden means, so that in the future these methods can be applied to further elucidate the toxic mechanism of BPA [13].

Increased prolactin levels, as a result of exposure to BPA and the detachment of a key role in autoimmunity associated with the production of anti-DNA antibodies, produce islet cell antibodies, thyroglobulin antibodies, thyroid peroxidase antibodies, adrenocortical antibodies, transglutaminase (in people with systemic lupus erythematosus—SLE), type 1 diabetes, Addison’s disease, Hashimoto’s disease, Addison’s disease, etc. [74,77]. 

Further long-term studies analyzing the development of autoimmune diseases linked to BPA exposure are needed.

### 2.6. Neuroendocrine System

US researchers at the 1991 Wingspread (Wisconsin) conference presented a classification of a new group of compounds that are endocrine-disrupting chemicals (including bisphenols and other parabens) [27,78,79]. It has been repeatedly confirmed that BPA (even in low doses) and some phthalates (known as endocrine disruptors) can mimic or interfere with hormone-signaling pathways. Examples of such hormones include estrogens, androgens, glucocorticosteroids, thyroid hormones and insulin, resulting in specific actions on tissue (e.g., within adipose tissue), reproductive organs, thyroid, liver, and pancreas [9,24,49,66,80]. By affecting thyroid development, BPA interferes with the whole thyroid system on many levels, such as acting as an antagonist of thyroid receptors in the cell membrane, or simply affecting the expression of thyroid hormone-related genes [9]. Undeniably, BPA exerts diverse and adverse endocrine effects on human physiology by binding to aryl hydrocarbon receptors [33,74]. Constant exposure to this xenoestrogen has deleterious effects on neurons, affecting their number and function [9,33].

Although all parabens are a well-known group of synthetic chemicals used as preservatives in a variety of products, there are still many issues related to their effects on human health. A large part of these unclear issues, often controversial, are still unexplained [27].

The impact of BPA during critical periods of development versus increased risk of neurodevelopmental disorders in various neurodegenerative diseases is clear. It cannot be ignored and further comprehensive studies should be pursued.

### 2.7. Cardiovascular System 

Also, some epidemiological studies confirm the association of BPA levels with the development of hypertension, cardiovascular or cardiometabolic diseases [13,17,18,24,37,81]. Unfortunately, it is widely believed that the cardiovascular system is highly susceptible to the destructive effects of BPA. For example, BPA has dose-dependent monotonic effects on Na+ and Ca 2+ channels, impairing cardiac electrophysiology [17,82].

BPA should be very carefully investigated for possible causes and links to systolic blood pressure (SBP), as Warembourg et al. showed that higher levels in the body can lower SBP [83]. Additionally, the NHANES study (2003–2006) in the U.S. showed that higher urinary BPA levels could also be potentially linked to ischemic heart disease in adults [84]. A study in Sweden of 1016 people over 70 years of age showed an association between serum BPA levels and atherosclerosis [85]. Peripheral artery disease (PAD), a subclinical measure of atherosclerotic vascular disease, was also associated with its concentration [17,86]. Unfortunately, this positive relationship between hypertension and BPA has been demonstrated beyond known common risk factors such as age, gender, BMI, smoking, race/ethnicity, diabetes, and total serum cholesterol [37]. A study in US boys [87] found an association between BPA and high diastolic blood pressure. Meanwhile, a study in mothers and children from Greece [68], showed that prenatal and child urinary BPA concentrations were not associated with blood pressure, cholesterol, leptin, adiponectin, and CRP levels at age 4.

Exposure to BPA adversely affects angiogenesis by stimulating vascular endothelial growth factor production. Consequently, it causes an increase in interventricular myocardial thickness and uncontrolled neovascularization [24], or the development of atherosclerosis, coronary artery disease, and dilated cardiomyopathy [17,18].

Thus, there is a need for further detailed studies evaluating the effects not only of the well-known risk factors but also of bisphenol A and its derivatives.

### 2.8. Metabolic and Other Diseases

There is a potential association of BPA levels with many diseases, including with obesity, type 2 diabetes, kidney disease (lower glomerular filtration rate), oxidative stress, lipid accumulation in the liver, non-alcoholic liver disease, the immune system (favoring asthma, bronchial hyperresponsiveness), frequent respiratory infection in childhood, autoimmune diseases [13,17,24,37,74,87,88,89,90,91,92,93,94]. Some reports/studies confirm that high levels of BPA (10–400 mg/kg) interfere with the normal functioning of pancreatic cells, at the same time causing complications in glucose regulation [24], and are positively associated with obesity [68]. Exposure to this xenoestrogen affects insulin resistance, glucose intolerance, and hyperinulinemia [95].

A study conducted in Dayton, Ohio, USA, in children (3–8 years old), showed that BPA concentrations in the urine of boys were associated with adverse liver effects [87]. Hence, any information obtained using NHANES data confirms the association between diabetes and higher urinary levels of this xenoestrogen [17]. A study in the RHEA pregnancy cohort (500 mother-child pairs) in Crete, Greece, found that creatinine-corrected BPA during pregnancy was negatively associated with BMI scores in children aged 1–4 years and with measures of obesity in early childhood. Yet, it was associated with increased BMI z-score, WC (waist circumference) and sum of skinfold thickness at age 4 [68]. Meanwhile, there is no clear answer or sufficient data to confirm the possible contribution of BPA exposure to the increased incidence of gestational diabetes [18].

Nonetheless, the link between BPA exposure and obesity is not fully confirmed because, as with many other conditions, it may be a component of many other factors.

### 2.9. Skeletal System

Recently, attention has also been drawn to the association of BPA levels with bone metabolism (impaired calcium and phosphate metabolism, decreased bone mineral density) [13,79,96]. It affects the function of two tissue populations: osteoblasts and osteoclasts, and in the case of the former group, inhibits their proliferative capacity. In the case of osteoclasts, its influence was associated with signs of apoptosis and inhibition of their maturation, thus affecting bone resorption. So, it is assumed that, unfortunately, it can disrupt bone homeostasis, both in the fetus and in adults. In vitro studies confirm the effect of BPA on human osteoblasts, so there may be inhibition of their development, various types of differentiation and, unfortunately, a decrease in the synthesis or mineralization of osteogenic genes (e.g., ALP—alkaline phosphatase). The authors clearly emphasize the need for further studies, including in vivo studies [80,97]. BPA affects bone mass loss, mainly by decreasing plasma calcium concentrations and inhibiting calcitin secretion, interferes with bone metabolism via the receptor activator of NF-B ligand (RANKL), and also affects the formation of bone morphogenic protein 2 (BMP-2) [97]. It is therefore clear that BPA can have degenerative effects on bone, both in adults and already in the fetus itself. Additionally, by affecting biological processes, it is linked to skeletal health, which may have implications for abnormal skeletal development and the pathogenesis of osteoporosis [79].

However, despite all of this, there is still a lack of evidence supporting the impact on all development in the human skeletal system, as this requires a multi-year and very long-term analysis. There is also a lack of research between BPA levels and the risk of fragility fracture or broader bone health in humans. It is very difficult to verify the effect of this one component on skeletal health, as we are too influenced by environmental factors.

### 2.10. Skin

It should be noted that the formation of BPA metabolites is observed in the dermis and receptor compartment rather than in the epidermis, often indicating a weaker detoxification process [13]. As BPA is commonly used in the production of thermal paper, it has been recognized as an important risk factor that cannot be ignored, especially for occupational groups (e.g., cashiers, print shop workers) [13,98]. Bernier and Vandenberg [99] estimated a BPA intake of 0.0511 μg/kg body weight/day in the school-age population as a result of exposure to this paper. Additionally, according to EFSA estimates, up to 0.059 μg/kg bw/day for dermal exposure [13,48]. With the most unfavorable estimate of frequent (150 times) contact with this paper, it can even penetrate 0.9543 μg/kg bw/day through the skin (and up to 3 times the concentration in urine in men) for occupationally exposed individuals [13,98]. Interestingly, it enters the body through absorption through the skin, but this process may be affected by the moisture content of the skin itself or fat extract [13,100]. When touching the receipt with a dry, clean hand, between 0.2 and 6 micrograms of BPA remains on the fingers, while when with greasy or wet fingers the amount increases tenfold, persisting on the skin for up to 2 h [101]. Some researchers have begun using hair to detect BPA concentrations in organisms, which is a much simpler sampling procedure, less prone to contamination, and consequently easier to store for long periods of time due to stability [100,102].

This non-dietary absorption of BPA into the body should be taken seriously and monitored especially in specific occupational groups.

### 2.11. Cancer

Unfortunately, several studies confirm the effect of even low levels of exposure to BPA on increasing the incidence of cancer itself, as well as already existing cancer [12,16]. The development of cancer may be influenced by epigenetic changes caused by exposure alone [16].

A number of studies confirm the association between BPA levels and the development of cancers, including prostate, breast, endometrial, ovarian, cervical, lung, testicular, acute myeloid leukemia, colorectal, liver, oral, pharyngeal, and thyroid cancers [12,13,16,103,104,105]. BPA affects the function of several signaling pathways, disrupting their proper functioning, within which it influences the development of low-grade breast, ovarian, liver, and endometrial cancers [12,97]. It is very well absorbed in adipose tissue and the striatum of the breast, so it may be responsible for the signaling pathways responsible for causing breast cancer [3,97]. For endometrial, cervical, and prostate cancers, BPA has also been identified as a key risk factor. Since BPA mimics estrogen it can also mimic its effects, unfortunately affecting tumor progression. Other studies confirm the link between environmental pollutants, including BPA, and impaired male germ cell development and testicular tumor development [12,105].

BPA can be involved in the induction of aggressive phenotypes in colorectal cancer, and its impact itself can vary widely. As the oral route is the first and most significant source for the delivery of BPA into the body, it is unfortunately also the first site of exposure. BPA has a high affinity for estrogen; then, unfortunately, the anti-cancer therapeutic process can be ineffective, due to the effects of its interaction with estrogen [12].

In the meantime, some studies suggest that BPA can accelerate glioma cell proliferation, but in a time- and dose-dependent manner. Importantly, it can increase the invasive and migratory capabilities of its cells, but also inhibit apoptotic processes [28,106]. More detailed functional, epidemiological, or even molecular studies focused on long-term observations and exposures are required for the analysis of any cancer.

## 3. Sources in Food/Packaging

Repeatedly, studies show that consumption of canned soup, coffee, canned and plastic-packaged foods, instant foods, canned foods, and foods provided by catering companies increase BPA concentrations [4,107,108,109]. As BPA’s oral route accounts for more than 90% of its total source, food and drinking water are important sources of exposure as well as ongoing control [7,13,16]. Epoxy resins are also used in drinking water pipes, from where it can migrate into drinking water [14,79]. The samples tested in food covering the average concentrations of BPA in different countries, sometimes differed from study to study. They may be the result of different food sample sizes, food processing, storage methods, laboratory methods used, breeding in different regions, etc. However, it is undeniable that its concentration in canned or tinned foods is higher compared to foods that are in other materials [13,79].

As there is also significant dermal contact of BPA as a result of contact mainly with receipts, the European Union has, as of January 2020, set a limit on the concentration of BPA in thermal paper at 0.02% by weight, further forcing other manufacturers to replace or even eliminate it [4]. Unfortunately, there is a widespread increasing presence of BPA in everyday items (e.g., food containers, food packaging film, toys, pens, kitchen utensils, medical products/devices, dental supplies, cosmetic supplies, personal care products, toothpaste, medications, helmets, cell phones, thermal paper (e.g., receipts, tickets, boarding passes), construction materials, CD/DVDs, and many others [1,3,4,11,13,19,20,21,22,23,27,38,78,79,80]. They are commonly used as plasticizers, solvents, fixatives, color developers (in thermal paper), cigarette filters, antimicrobial agents, or commonly used preservatives [13,68,80,110].

With increasing temperature, the migration of BPA as well as its derivatives also increases, such as into canned tomatoes, canned seafood with high lipid content, soft drinks and energy drinks, beers, canned fish and meat products. And in water packaged in PC bottles, the percentage of BPA accounted for 60% after 1 year of storage in these packages. Also, its content is significantly higher in damaged cans, compared to undamaged ones [6,16].

Albeit longer and more in-depth studies will be required to observe the possible toxic effects released from dental/medical materials in general.

## 4. Environment

Frankowski et al. [4] noted that particular bacterial species that degrade one bisphenol do not degrade all other bisphenols. As the authors point out, simply replacing legally regulated BPA with other bisphenols can result in increased environmental pollution until bacteria adapt to the new nutrients. Frankowski et al. also point out that in addition to their own, other results available in the literature are contradictory, and biodegradation of BPA in water can last up to 5 days. The lower biodegradation of BPA in water may be determined by the lower bacterial biodiversity and the presence of two or more bacterial strains, not just one. Again, it is worth noting that different bacterial strains act on different bisphenols in different ways. Other bisphenols, unfortunately, can persist in water for up to several weeks at a time, and the activity of the microorganisms can be negligible. BPA and BPF are completely primary biodegradable, supporting the ability to remove these compounds from the environment, and others were poorly biodegradable. The authors again pointed out that the dominant bacterial species after the initial biodegradation may not be capable of further degradation [4,111,112]. Thus, the effectiveness of these microorganisms may be negligible and furthermore, very uncertain.

In an experiment by Zielinski et al. [111] lasting for 420 days, the concentration of BPA in wastewater was analyzed in great detail. The authors suggest that whether the biodegradation of BPA and its derivatives is direct or indirect, the performance of the bioreactor itself depends on the diversity of the different microbial groups. Meanwhile, testing of wastewater at an experimental site in Langenreichenbach, Germany, showed that bisphenol A had the lowest removal efficiency on average [56]. The process itself is quite challenging yet worthy of further investigation. 

It is worth noting that BPA degrades rapidly in surface waters. In contrast, very slow degradation can occur under certain environmental conditions. BPA is not considered to have a high bioaccumulation capacity in living organisms. Yet the environment, e.g., water, air (through contaminated soil, not food-contact materials) is not considered the main route of exposure. Meanwhile, people may be exposed to BPA indoors resulting from leaching of BPA from polycarbonate plastic products and contaminated dust, dust [7,10,13,24,44,79,111].

Ohore and Zhang [24] reported concentrations of BPA in surface water, e.g., in China 96 ng/L, Korea (5.7/102 ng/g), USA (5.1 ng/g dw), and Japan (8.2 ng/g), indicating a strong and urgent need for its disposal. Meanwhile, more intensive consideration has recently begun to be given to methods of BPA removal, such as in water treatment plants, using advanced oxidation processes or general biodegradation with biological treatment (e.g., enzymes). Chemical methods rely on hydrogen bonding or hydrophobic interactions [10,24,87,111]. Unfortunately, the efficiency of BPA removal from municipal wastewater treatment plants most often fluctuates around 84%, which can depend on operating parameters, the scale of contamination, the methods used, their validation, form, and the length of time the methods have been used [87].

There is also still a lack of clear information on the combined effects of BPA with other pollutants and sources from the environment [13,111]. It is worth noting that high temperatures lead to the release of more BPA not only into beverages and food but also into the environment [16,79]. This most toxicity-laden compound is being replaced by analogs of the so-called NextGen bisphenols, also known as “BPA-free” making them unfortunately ubiquitous [18,82]. 

So far, the use of other bisphenols (F, A, AF, B, C, E, F, G, M, P, S, Z) is rare. BPAF is an analog of BPA, which is not yet so widely used in the environment. BPF, unfortunately, has similar endocrine activity to BPA, but is still not considered ubiquitous and a source of pollution. BPAF, which is an analog of BPA, is not yet as widely distributed in the environment as BPA, although it is responsible for the chemical stability as well as the thermal stability of the compound. It is used in the manufacture of electronics and optical fibers, and in the processing of fluoroelastomers. These three main bisphenols, unfortunately, are its natural substitutes, and are ideal for bypassing various restrictions and regulations to keep production going [4,10,18]. The seven best-known bisphenols (AP, S, Z, B, A, F and AF) have so far been detected in lake water in China, sludge, sewage and sediment, food, bottled water, personal care products, thermal parathas, and confined spaces [18].

Advanced methods of disposing of BPA from the environment may be of concern due to the high cost and, once again, to verify their effectiveness. It is worth emphasizing that such methods should be used to remove not only BPA itself but also its derivatives and, consequently, many other toxic substances.

## 5. Guidance from Various International Organizations

Regulatory opinions on the safety of BPA in packaging manufacturing still vary widely. Current regulations proposed by the European Union (Regulation (EC) No. 1223/2009; (EC) No. 2023/2006; (EC) 1907/2006; (EC) No. 1935/2004), among others, are still of great concern with regard to BPA, as they do not take into account the latest knowledge on, for example, dose–response relationships, mixtures resulting from the joint use of products, and exposure effects occurring at different stages of human development [110]. Meanwhile, Commission Regulation (EU) No. 10/2011/EU2 on plastic materials and articles intended to come into contact with foodstuffs reduced the migration limit from 3 mg/kg to 0.6 mg/kg in 2002 to 0.005 mg/kg in 2015 [18,44,113]. 

The European Food Safety Authority (EFSA), after assessing the risks of BPA, proposed a tolerable daily intake (TDI) of 4 µg/kg bw/day [114], and in 2023 revised the TDI recommendation to 0.2 ng/kg bw/day, reducing 20,000 times what it was before [44,113]. Both EFSA and the Food and Drug Administration (FDA) believe that BPA levels currently in food do not exceed the limits causing adverse effects [4,44]. An EFSA expert panel confirmed that there are health concerns from dietary exposure to BPA, among others, in all age groups of the general population [14,44]. It is worth noting that the EFSA panel, estimating dental sealants, estimated that they amounted to less than 0.001% of total BPA exposure [48], so according to their opinion there is little reason for concern about potential health risks [6,13].

The European Environment Agency specifically highlights the harmful effects of BPA on both the human body and the environment. The European Commission, in a new proposal (likely to be ready in the first quarter of 2024), intends to propose a ban on BPA and other bisphenols in food contact materials in favor of zero contamination [14]. 

It is interesting to note that the European Medicines Agency (EMA) and the German Federal Institute for Risk Assessment (BfR) have expressed rather divergent scientific opinions on how to determine TDI values for BPA, albeit agreeing over its toxicity [14,113,115].

It is interesting to note that based on the data of negative health effects of BPA, Canada in 2008 became the first country to classify it as a toxic chemical, banning its use in infant products. Following their example, these bans have been introduced in the US, some European countries, and Asian countries [13]. The Food and Drug Administration (FDA), after analysis and in-depth studies to assess the potential risk of BPA in the diet, is supporting industry efforts to stop the production of infant bottles containing the above xenoestrogen. It is now paying special attention to its alternatives in the production of infant can liners. Unfortunately, the FDA is not changing its mind about the use of infant formula or food. It believes that the benefits of good nutrition may outweigh the potential health risks [116].

In 2010, experts from the Food and Agriculture Organization (FAO) and the World Health Organization (WHO) estimated that BPA from soil/dust consumption could be as high as 1.0 × 10^−4^–0.03 µg/kg bw/day for the general population [13,42]. The World Health Organization panel did not advocate additional regulations on BPA and its further evaluation [116].

Meanwhile, the Australian and New Zealand Food Safety Authority did not consider the health risks of consuming BPA, mainly by infants using baby bottles containing the xenoestrogen. A 2016 study in Australia confirmed low dietary exposures among consumers and are within acceptable, safe limits. In New Zealand, this issue is still unregulated and many companies use it further in the manufacture of baby products [116,117]. 

The UK’s Committee on Toxicity of Chemicals in Food, Consumer Products and the Environment (COT) is currently developing an interim paper on the toxicity of BPA. This is because it has concluded that the weight of evidence does not support EFSA’s conclusions or a TDI as low as they have set [118]. 

Denmark, Sweden and Belgium have set restrictions on the use of BPA in food packaging for children aged 0 to 3 years, while Sweden has limited it only to products in varnishes and coatings for food packaging for the same age group. Austria has only restricted the use of this xenoestrogen in pacifiers and bottles since October 2010 [17,18]. France, meanwhile, has banned the use of this xenoestrogen in all materials in contact with food (LOI No. 2012-1442 from 24 December 2012). Among the many numerous meta-analyses conducted in different regions of the world, it has even been detected in parts of rural Africa with higher industrial levels [18]. 

Our everyday stuff is made of plastic (including polyester as the main one), so BPA can be everywhere, including even on clothing. Clothing manufacturers unfortunately do not have to provide information on the label about the chemicals used to produce the fibers or treat the resulting fabric, so BPA is common.

Thus, such divergent guidelines and the lack of a common policy for all institutions, companies or even consumers can create confusion and production freedom. Unfortunately, to the detriment of our health. In conclusion, it is worth noting that almost all US and European institutions see the problem that is the use of bisphenol and its toxic effects at every level of the body, yet their guidelines are the most stringent of all those found around the world. Here, the problem arises, due to, for example, almost no guidelines in less developed countries/regions/continents of the world, which are the source of production of products with BPA. Thus, the creation of a regulatory framework should not only be respected at the European or North American level, but also jointly created on other continents. 

As the authors pointed out, all these global or regional regulations have led to BPA being increasingly replaced by other bisphenols, known as “BPA-free” or next-generation (NextGen bisphenol) products. Some EU countries have proposed a detailed study of the activities of alternatives to BPA and regulating their BPA-like status (Plastics Europe Citation 2022) [18,119]. This requires numerous clinical or experimental studies verifying their safety at every level. Thus, it is difficult to verify and give universal standards/regulations until the WHO explicitly imposes them for the whole world. 

The Carcinogen Identification Committee on 14 December 2022 declined to add BPA to the 65 List of Carcinogens, acknowledging that the epidemiological evidence available in the literature is insufficient to assess BPA’s carcinogenicity due to limitations such as insufficient exposure assessment [32]. 

In the U.S., the Consortium Linking Academic and Regulatory Insights on BPA Toxicity (CLARITY-BPA) program was designed to examine the full range of potential health effects of BPA exposure in rats in order to completely dispel doubts. Where even the FDA has stated that after an extensive review of the literature on low doses of BPA (and here it is still unclear what kind because there are differences in interpretation), they are unable to construct a reliable or logical comprehensive toxicological profile for this xenoestrogen. Thus, they cannot explain all the claimed health effects of BPA, mainly because of the inconsistencies that currently exist in the literature. This project had reduced many potential sources of bias, randomly and without labeling samples were sent to the lab from all over North America [120,121]. A detailed description of the experimental procedures, results and interpretation is included in the report, so any interested reader can find more details there.

## 6. Prevention

Primary prevention involves taking measures to ensure that our environment, including everyday objects, are safe for the general population. A very important and fairly well-regarded measure is self-intervention. Namely, education of the general public, as well as health professionals and decision-makers, can result in broader actions to reduce exposure. Examples of such educational activities could be films, games, leaflets, meetings, lectures or other spots promoting the reduction of BPA-containing everyday products. Following are examples of activities aimed at individuals:

- Choose glass, porcelain or steel packaging instead of plastic, especially for hot foods;
- Try to choose only BPA-free containers;
- Instead of canned products, use dried, such as seeds;
- Do not choose plastic containers with recycling codes at the bottom of three or seven, they may be made of BPA;
- Reduce your use of canned foods and beverages;
- If you can, use BPA-free baby bottles;
- Use wooden or other safe BPA free toys;
- Avoid heating BPA products in the microwave or dishwasher, as it will seep into food;
- Read the labels and composition of each product;
- Choose natural, organic products made from safe and non-toxic ingredients;
- Do not accept paper receipts, bills or tickets;
- If you work in a store wash your hands at every possible opportunity;
- Check your products online to see if they contain BPA and can be avoided, including medical products (ask your doctor or other medical professional);
- Parents can ask schools to give their children educational lessons to get them in the habit of making good choices from an early age;
- Change your exercise clothes, as there is a chance they may contain polycarbonate and when exercising in warm weather, BPA will be absorbed faster through the skin.

Meanwhile, action in the manufacturing and packaging sector could reduce its health effects in the broadest way with the lowest burden [110,120,122,123]. The harmful health effects of BPA have caused great concern among many scientists and the public, which has at least resulted in a ban on the sale of plastic products used on infants and young children [3,79]. Biomonitoring data (e.g., levels in urine, blood or hair samples) can help researchers plan and conduct studies on BPA exposure and its health effects [14,38]. In the 11 European countries that participated in BPA biomonitoring, exceedance levels ranged between 71% (Switzerland), 83% (Germany), 86% (Denmark), 90% (Finland), 96% (Croatia), 98% (Iceland, Czechia), 99% (Poland), and 100% (France, Luxemburg, Portugal) [14]. 

Making a brave hypothesis, one could suppose that by discovering these various types of aberrations at the cellular level, a marker could be invented, resulting in the preparation of an entire testing protocol. This could facilitate a faster procedure for test sensitivity (without unnecessary sensitivity and from the point of collection (even non-invasive) through simple methodology, analysis and prevention focused on future exposed workers. 

It is absolutely necessary to arouse greater consumer interest, in terms of the safety of cosmetic, food and medical products available on the market, as it is an important factor in developing the best strategies for preserving these products [79,100]. Thus, it is necessary to at least replace or minimize materials with any content of BPA and its derivatives in the packaging industry with food-grade films and coatings, replacing them with biodegradable, human-friendly materials [6,79].

Meanwhile, it should be emphasized that the most responsible people should be the caregivers who purchase the products in question and use them on an ongoing basis.

## 7. Conclusions

Bisphenol A is the most widely known chemical and perhaps even the most researched by virtually all international or national organizations, but it is, nonetheless, still controversial. Despite the huge number of population studies covering BPA and its adverse effects, the results are still contradictory. This inconsistency may be influenced by a number of results, such as sample collection methods, individual characteristics or the type of research used.

In general, BPA levels are still too high as a result of biomonitoring and pose a potential threat to public health. It is beginning to be widely recognized that future toxicity studies should focus on molecular biology and the assessment of human exposure to BPA, as well as its substitutes. The effects of its exposure to humans still require years of observation, extensive research and answers to many questions. The combination of BPA-induced health effects along with environmental contaminants absolutely must be confirmed by future, thorough, extensive research. Further and in-depth studies assessing the risks of BPA substitutes are equally important, as epidemiological studies confirming their unequivocal impact are still lacking. 

As already emphasized several times in this work, it should be kept in mind that it is difficult to point with certainty to one xenoestrogen that is responsible for all diseases and dysfunctions in the body, as we are surrounded by other and harmful ingredients.

It is necessary to continue to deepen the knowledge and interest of consumers around the world in order to make rational purchases as well as future choices, not only consumer ones.
